# Preresonant Coherent IR-hyper-Raman Spectroscopy of FAPbBr_3_

**DOI:** 10.1021/acs.jpclett.6c02047

**Published:** 2026-09-11

**Authors:** Ryan P. McDonnell, David P. Lafayette, Daniel D. Kohler, Jason M. Scheeler, Jalianet Román-Matías, Willa Mihalyi-Koch, Song Jin, John C. Wright

**Affiliations:** Department of Chemistry, University of Wisconsin-Madison, Madison, Wisconsin 53706, United States

## Abstract

Electron–phonon coupling is believed to influence many properties in lead halide perovskites (LHPs), including stability and photoefficency. Identifying the vibrational coordinates that compose excitonic potential energy surfaces in lead halide perovskites would prove instrumental for identifying the origin of these properties. Hyper difference frequency generation (HDFG) spectroscopy, which uses infrared and hyper-Raman transitions to measure coherent spectra and dynamics, has potential to resolve these couplings. As a first step toward the use of HDFG in identifying the vibrational coordinates and resolving these couplings, we measure the preresonant HDFG spectrum of FAPbBr_3_, a centrosymmetric 3D lead halide perovskite. The preresonant spectrum is found to be in agreement with FT-IR spectra and resolve A-site cation vibrations, suggesting that HDFG can be a useful tool for mapping out resonance trends that can inform on electron–phonon coupling, similar to those found in resonance Raman and hyper-Raman spectroscopy. This new approach to resolving vibrational spectra in materials systems is extendable to both centrosymmetric and noncentrosymmetric perovskites.

## Graphical abstract

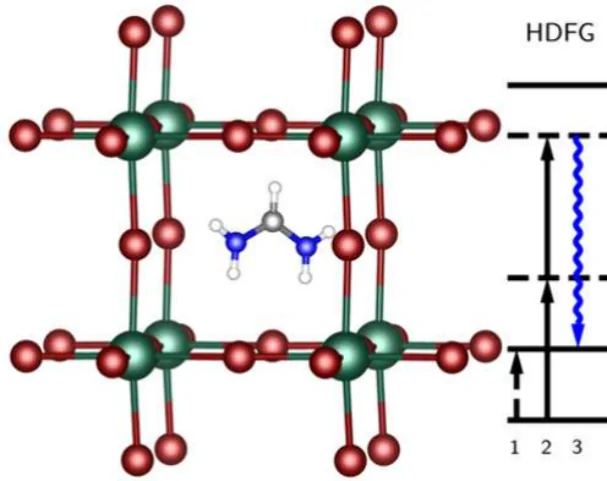

Coupling between electronic and vibrational degrees of freedom dictate charge transport properties in many 2D and 3D semiconductors.^1–3^ Such couplings are believed to be highly consequential in hybrid organic–inorganic lead halide perovskites (LHPs),^4–14^ which have the chemical formula APbX_3_, where A is usually a small, typically amine-based, organic cation such as formamidinium (FA = [HC­(NH_2_)_2_]^+^) and X is a halide.^15,16^ The PbX_3_ moiety forms a negatively charged octahedral network, and the A cation occupies the inter-octahedral cavities (the “perovskite cage”) by intermolecular forces (Figure 1).^17,18^ The organic A-site cation introduces disorder to the perovskite: the A-site cation has restricted rotational motion, and the ammonium (−NH_*n*_^+^) moieties can hydrogen-bond at multiple sites within the octahedral cage.^11,19–22^ This interplay of rotation and bonding strongly influences the properties of LHPs, playing a role in order–disorder phase transitions,^23–25^ structural stability,^26^ and potentially charge carrier dynamics through screening and/or self-trapping.^4,27–30^

**Figure 1:**
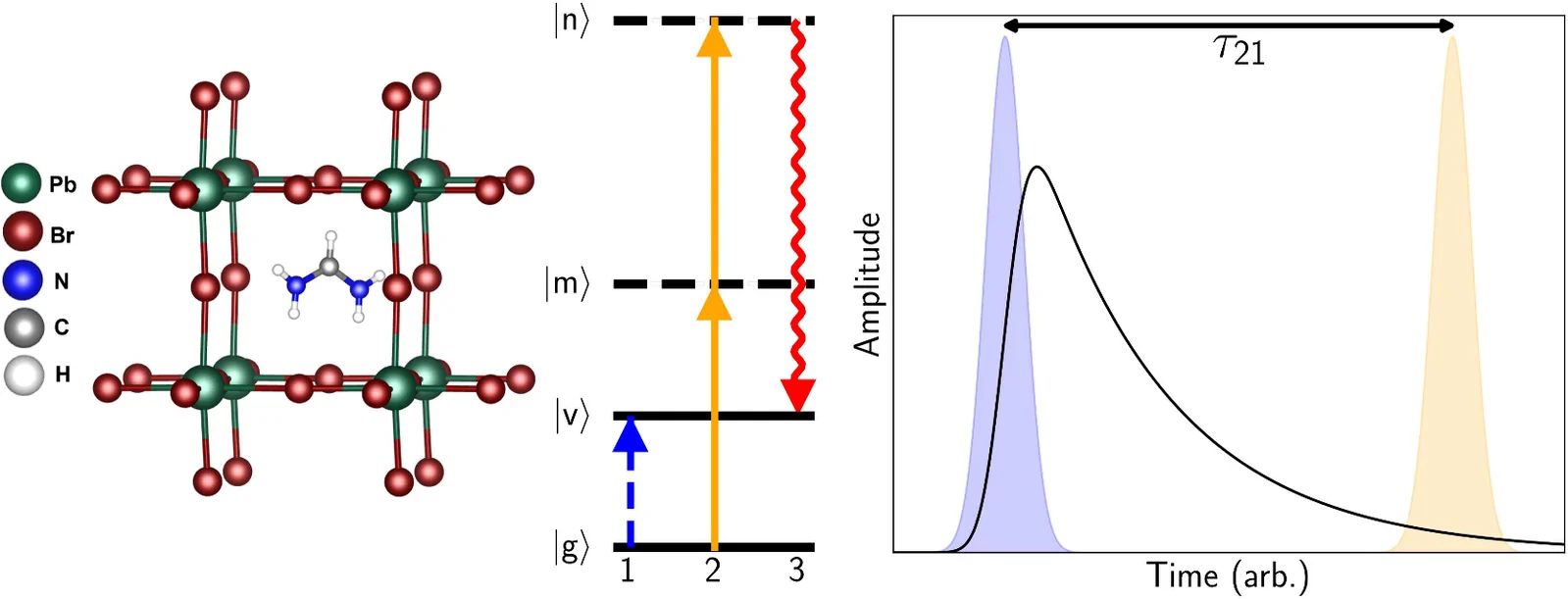
Schematic of the preresonant HDFG experiment on FAPbBr_3_. (left) Schematic of a FAPbBr_3_ unit cell, adapted to portray the FA cation. (center) Wave mixing energy level (WMEL) diagram of HDFG.^45,54^ |*g*⟩ is the ground state, |*v*⟩ is a vibrational mode, and |*m*⟩ and |*n*⟩ are virtual states. The dashed (solid) arrow indicates propagation of the bra (ket)-side density matrix element. (right) Pulse arrangement of an HDFG experiment. The IR pulse (blue, ω_1_) excites a vibrational mode |*v*⟩, creating a vibrational coherence |*g*⟩⟨*v*| (black trace) that undergoes free induction decay (FID) and dephases with time constant *T*_2_ (cf. eq 1). A two-photon interaction (orange, 2ω_2_) stimulates Stokes hyper-Raman scattering to upconvert the vibrational FID and is homodyne-detected at frequency 2ω_2_ – ω_1_. The time between the infrared interaction (at time τ_1_) and the two-photon interaction (at time τ_2_) is τ_2_ – τ_1_ ≡ τ_21_ = −τ_12_.

There is growing evidence that A-site cation vibrational modes that participate in short-range interactions with the perovskite lattice strongly influence the potential energy surface of excitons.^4,11^ Lattice vibrations are known to be coupled to excitons in LHPs.^5,6,13,31–34^ Nonlinear spectroscopic investigations of LHPs have revealed signatures of anharmonicities involving A-site cation and lattice vibrations.^22,35^ Resonance Raman spectroscopy on 2D and 3D LHPs have shown intensity enhancement of both lattice and A-site cation vibrations, supporting that some ground-state vibrational modes serve as excitonic coordinates.^4,36^ Vibrations associated with the A-site cation are usually normal modes that can be identified by routine quantum chemical calculations.^21,37^ Evidence from a variety of spectroscopic techniques and perovskite systems suggests that excited-state structure is governed by a complex interplay of ground- and excited-state vibrations. By identifying the A-site cation vibrations, and thus molecular motifs, involved in electron–vibration coupling schemes, cation selection could be intelligently tailored to specific applications. As a result, developing a general picture of how A-site cation vibrations contribute to excitonic coordinates is desirable.

Prominently used in molecular spectroscopy, an approach to explore the relationship between ground- and excited-state coordinates is frequency-dependent resonance Raman spectroscopy.^38–41^ However, the photoluminescence from LHPs, known to overwhelm desired optical response,^42,43^ can contaminate frequency-dependent Raman spectra, making systematic examination of these relationships by spontaneous Raman spectroscopy difficult. An alternative approach to measure resonance trends is to use coherent nonlinear spectroscopies,^44,45^ whose output can be isolated against competing background processes such as photoluminescence.^46^ Coherent nonlinear spectroscopy can naturally incorporate internal standards via nonresonant signals from the bath and/or substrate, allowing examination of frequency-dependent resonance trends.^47–49^ A recently developed coherent nonlinear spectroscopy that shows promise for investigating electron–vibration-type coupling involving A-site cation vibrational modes in LHPs is hyper difference frequency generation (HDFG) spectroscopy.^50,51^ Diagrammed in Figure 1, HDFG uses an infrared pulse (ω_1_) to create a vibrational coherence that is then upconverted through a two-photon interaction with a near-infrared (NIR) pulse (ω_2_).^45^ The upconversion stimulates Stokes hyper-Raman scattering with a coherent output at frequency 2ω_2_ – ω_1_.^50,52^ Since HDFG response is dictated by infrared and hyper-Raman selection rules and all IR active modes are hyper-Raman active,^53^ HDFG resolves infrared active vibrations.

Preresonant Raman or hyper-Raman response is an essential first step in identifying how ground-state vibrations are related to different electronic surfaces.^39,55^ In this report, we demonstrate preresonant, mixed-domain HDFG spectroscopy of a FAPbBr_3_ single crystal. With 2ω_2_ = 15400 cm^–1^, roughly 3000 cm^–1^ detuned from the band edge,^56^ we resolve HDFG response from the *ν*(NCN) stretch of FAPbBr_3_. The coherent HDFG response is contaminated by nonresonant signals at pulse overlap, but by changing the time delay between the infrared (ω_1_) and upconversion (ω_2_) pulses,^57^ the vibrational signal is isolated. The *ν*(NCN) stretch exhibits a dephasing time of *T*_2_ = 0.8 ± 0.1 ps, in excellent agreement with the infrared spectrum. These results demonstrate that HDFG is viable for investigating the excitonic coupling in FAPbBr_3_ and other LHPs.

To perform preresonant HDFG, coherent output from a FAPbBr_3_ single crystal in the **k**_3_ = 2**k**_2_ – **k**_1_ phasematching direction was recorded as the input infrared frequency and time delay was scanned. The pulsewidths are roughly 1 ps. The monochromator tracked the output frequency at 2ω_2_ – ω_1_ as the infrared laser frequency (ω_1_) and time delay between ω_1_ and ω_2_, τ_12_ ≡ τ_1_ – τ_2_ were scanned; when τ_12_ is negative, ω_1_ interacts with the sample first, allowing direct measurement of vibrational free induction, diagrammed in Figure 1. Since the HDFG output color is redder than FAPbBr_3_ photoluminescence (maximum emission ∼18200 cm^–1^),^11^ contamination from excitation scatter and photoluminescence is minimized.

Figure 2 explores the HDFG spectrum of the *ν*(NCN) stretch and the delay between the infrared and two-photon interactions, τ_12_. When the two-photon interaction occurs well after the IR excitation (τ_12_ ≤ −1 ps), the HDFG spectrum shows a sharp feature centered about ∼1720 cm^–1^, indicative of the FA cation *ν*(NCN) mode. Since the data is collected by having the monochromator track the frequency 2ω_2_ – ω_1_, undesired artifacts from leakage processes or photoluminesence will be detected. Indeed, the spectral response ∼1800 cm^–1^ is suspected to arise from poorly suppressed leakage contributions from the optical parametric amplifiers (OPAs) used in this work, as its intensity is lessened in HDFG spectra collected at 2ω_2_ = 15000 cm^–1^ (Figure S1). Note that the negative signal is found at positive and negative delay times (Figure S1); the resonance is identified through a combination of the free induction that persists at negative delays and dispersive lineshape at 1720 cm^–1^ (vide infra) at zero delay. The ∼1620 cm^–1^ mode (δ­(NH_2_)) of FAPbBr_3_, seen in both IR (Figure 3) and Raman spectra,^11^ is notably absent in the HDFG spectrum. However, given its weakness in the infrared spectrum, it is not expected to be a strong HDFG mode.^45,51^ While some signal appears around 1620 cm^–1^ around τ_12_ = 0, the structure seems inconsistent with δ­(NH_2_), and its absence for long τ_12_ delays makes it an unlikely contribution to the overall HDFG response.

**Figure 2:**
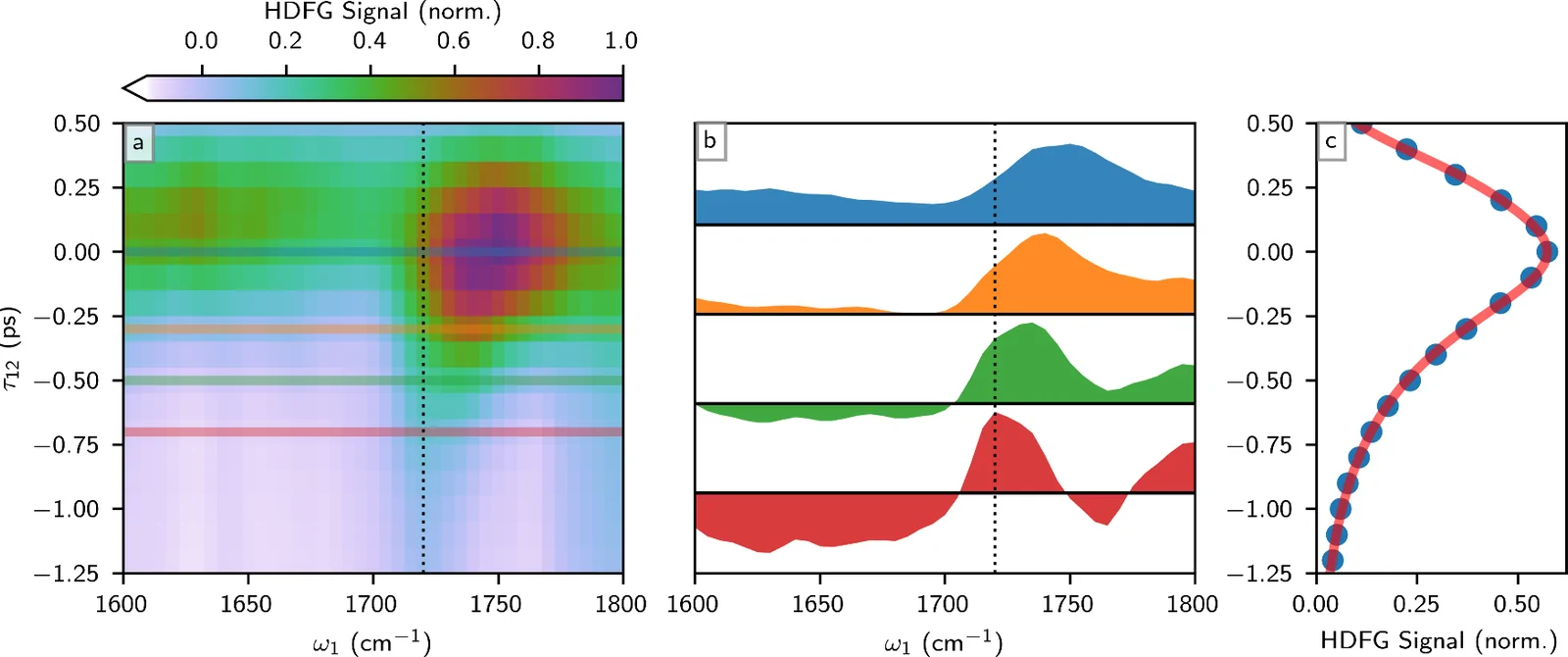
HDFG response from single crystal FAPbBr_3_, where the monochromator tracks output at ω_m_ = 2ω_2_ – ω_1_ and ω_2_ = 7700 cm^–1^. (a) Frequency-time response in the 1600–1800 cm^–1^ region. The spectrum in (a) has been smoothed and leveled to account for H_2_O and other effects on pulse propagation, which results in “negative” intensities at some frequency and delay values. The two-photon interaction frequency is 2ω_2_ = 15400 cm^–1^. (b) 1D slices of the HDFG response in (a). Colors correspond to the horizontal slices in (a), indicating the time delay. (c) Dynamics of the HDFG response at ω_1_ = 1720 cm^–1^. The thick red line overlaying the experimental response (blue dots) is a fit to eq 3 with *T*_2_ = 0.8 ± 0.1 ps.

**Figure 3:**
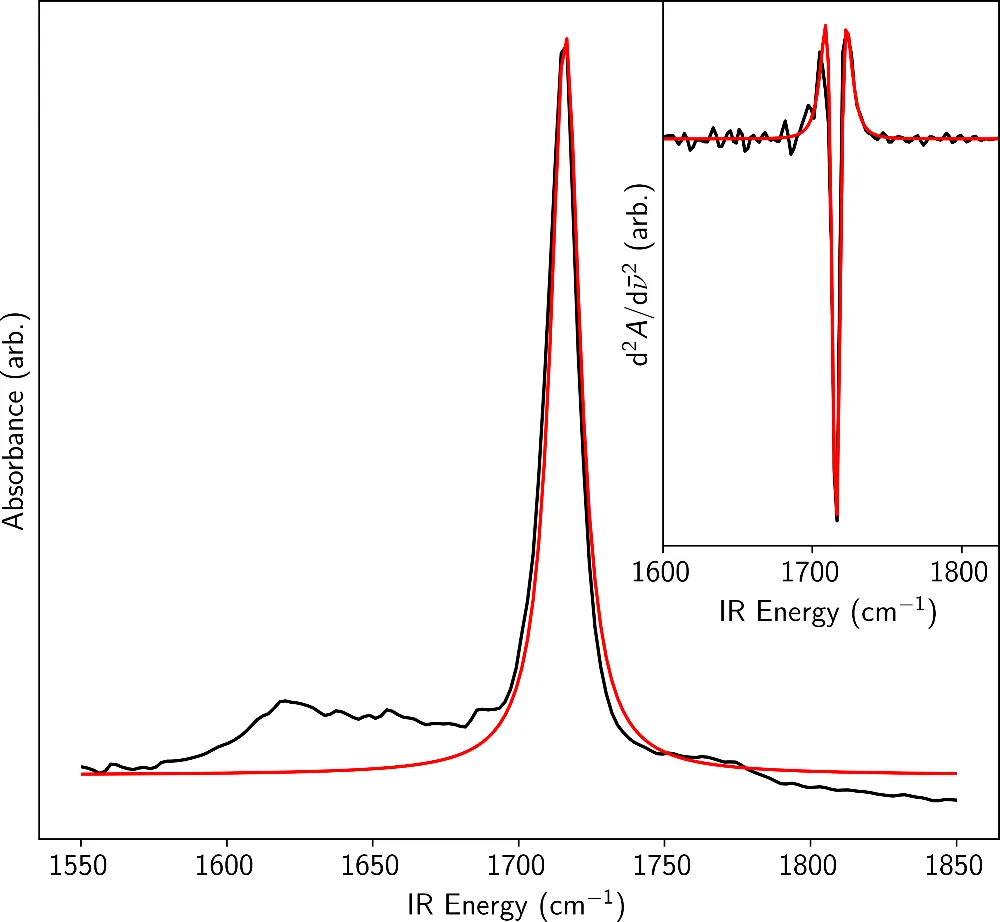
ATR-FT-IR spectrum of a FAPbBr_3_ single crystal. Black: recorded spectrum. Red: Lorentzian lineshape fit to *ν*(NCN) with *T*_2_ = 0.87 ps. The inset shows the second derivative spectrum of the data and the fit. Fitting the spectrum and its second derivative simultaneously is useful for isolating resonant features.^67^

As the delay value approaches full temporal overlap (τ_12_ = 0), the HDFG lineshape near ω_1_ = 1720 cm^–1^ becomes dispersive and the peak position blueshifts. This lineshape evolution arises due to interference between the resonant HDFG response and background nonlinear response.^45,47,48^ The background response is nonresonant and only persists at delays where the pulses have appreciable temporal overlap. In contrast, the resonant vibrational signal follows the free induction of the vibration. At temporal overlap, the roughly static background response interferes with the frequency-dependent HDFG resonance, giving the dispersive lineshape,^47^ an indicator of a resonant vibrational HDFG response. Unlike three-pulse photon echo and related measurements,^58,59^ singly resonant HDFG cannot unambiguously identify whether a vibration is homogeneously or inhomogeneously broadened,^60^ similar to vibrational sum frequency generation (vSFG) and singly resonant coherent (anti-)­Stokes Raman spectroscopy.^61,62^ Coherent dynamics extracted from HDFG measurements should therefore align with those found in an FT-IR spectrum.^45^

The free induction lifetime can be extracted through fits using the typical response function formalism.^63^ Invoking the Placzek approximation,^64^ where the nonresonant pulse (ω_2_) interacts instantaneously with the sample (i.e., virtual states),^45^ and using the pulse sequence in Figure 1, the singly resonant HDFG response function *R*_HDFG_^(3)^ is

1 $$R_{\mathrm{HDFG}}^{\left(3\right)}\left(t_{2},t_{1}\right)=-\frac{i}{\hbar }\theta \left(t_{1}\right)\left\langle \beta \mu \right\rangle \;\exp\left(-\frac{t_{1}}{T_{2}}\right)\;\delta \left(t_{2}\right)\rho_{gg}$$

where ρ_*gg*_ is the ground-state population prior to excitation, β and μ are the hyper-Raman hyperpolarizability and infrared transition dipole, respectively, and the angular brackets indicate orientational averaging. In eq 1, the infrared pulse is assumed to be resonant, so dynamics from detuning is neglected.^61^ The tensorial nature of *R* ^(3)^ and its components is suppressed. In this expression, exponential dephasing (i.e., Lorenztian lineshapes) is assumed, typically appropriate for homogeneous vibrational dephasing.^65^ To account for background response when the input pulses are temporally overlapped, the total response function is

2 $$R^{\left(3\right)}\left(t_{2},t_{1}\right)=R_{\mathrm{HDFG}}^{\left(3\right)}\left(t_{2},t_{1}\right)+Ae^{i\phi }\delta \left(t_{2}\right)\delta \left(t_{1}\right)$$

where *A* is a real valued constant and ϕ is a phase. The total response function is used to generate a third-order polarization that is used as a source in the Maxwell equations to describe the launched HDFG field.^63^ The time-integrated intensity of the launched field is given by^45,66^

3 $$I_{\mathrm{HDFG}}\left(\tau_{12}\right)\propto {\left|\int_{-\infty }^{\infty }dt\;{\left(E_{2}\left(t+\tau_{12}\right)\right)}^{2}\int_{-\infty }^{\infty }dt_{1}\;\left[Ae^{i\phi }\delta \left(t_{1}\right)+B\exp\left(-\frac{t_{1}}{T_{2}}\right)\;\theta \left(t_{1}\right)\right]E_{1}\left(t-t_{1}\right)\right|}^{2}$$

where *E*_1_ is the infrared input field envelope, *E*_2_ is the near-infrared input field envelope, and *B* accounts for the coefficients in eq 1. We assume Gaussian envelopes and extract our excitation pulsewidths from cross-correlation measurements. Fitting the transient at the *ν*(NCN) frequency to eq 3 (cf. Figure 2c) provides a dephasing time *T*_2_ = 0.8 ± 0.1 ps. This dephasing time should correspond to the homogeneous vibrational linewidth 2Γ_*vg*_, where $\Gamma_{vg}=\frac{1}{2\pi cT_{2}}$ , in which *c* is the speed of light.^61^ HDFG spectra taken at a different two-photon interaction color, 2ω_2_ = 15000 cm^–1^, provide similar results (Figure S1).

We can now compare our coherent vibrational dynamics to other dynamics of the *ν*(NCN) vibration. The FAPbBr_3_ infrared spectrum (Figure 3) is well-fit by a Lorentzian lineshape, consistent with 2D-IR experiments on the halide analogue FAPbI_3_.^21^ We extract *T*_2_ = 0.87 ps (cf. Figure 3), in excellent agreement with the HDFG dynamics. Despite the single-crystal nature of our sample, this linewidth matches that of the thin-film morphology.^11^

The coherent dynamics also provides context for the population decay rate, which has been previously reported in FAPbI_3_.^21^ For a fully homogeneous oscillator, the dephasing time *T*_2_ is related to the population lifetime (*T*_1_) and pure dephasing time *T*_2_^*^ by^68^

4 $$\frac{1}{T_{2}}=\frac{1}{2T_{1}}+\frac{1}{T_{2}^{*}}$$

Fluctuations in the distribution of vibrational frequencies, usually assigned to interactions with the thermal bath and anharmonicites,^69,70^ give rise to pure dephasing.^71^ In the case of *ν*(NCN), fluctuations likely arise because the FA cation tumbles in the lattice and samples multiple sites.^11^ If we assume that the population relaxation of *ν*(NCN) does not significantly change between FAPbBr_3_ and FAPbI_3_, as has been done previously,^11^ we can then take the population relaxation of *ν*(NCN) from that found in FAPbI_3_, *T*_1_ = 2.8 ± 0.5 ps,^21^ and find the pure-dephasing contributions to be *T*_2_^*^ = 0.9 ± 0.1 ps. The fact that the pure dephasing of *ν*(NCN) is the dominant contribution to the total dephasing time is not unexpected, given the rapid reorientation of the FA cation.^11,21^ Similar results have been found for high-energy vibrations of spacer cations in 2D perovskites.^71^

The present work has only isolated preresonant HDFG response from FAPbBr_3_, which is not an explicit measure of the desired electron–phonon coupling information that HDFG could provide and, in practice, duplicates the information accessible through ATR-FT-IR spectroscopy. Due to limitations on available excitation frequencies from the OPA used in this work to provide the ω_2_ excitation, the band edge of FAPbBr_3_ was not accessible. Frequency-dependent HDFG experiments which scan ω_2_ across excitonic resonances give the hyper-Raman excitation profile associated with the vibration excited by ω_1_.^45,51^ Quantitative assessment of the hyper-Raman excitation profile for different A-site cation vibrations can identify how ground-state vibrations are related to the allowed electronic and excitonic transitions in numerous LHP systems. Such experiments would be best performed on two-dimensional LHPs, as their high degree of tunability and relatively large transition dipole moments provide ideal systems for exploring the interplay of electron–vibration coupling in LHPs.^8,72–74^

While the present measurements highlight the potential of HDFG spectroscopy for investigating resonance hyper-Raman profiles of metal halide perovskites, they also more generally highlight the viability of controlling vibrational coherences in FAPbBr_3_ and other LHPs. Manipulating vibrational coherences is key when investigating vibrational anharmonicity involving high-frequency vibrations by multifrequency nonlinear spectroscopy.^35,75,76^ Some pulse sequences, such as those in doubly vibrationally enhanced (DOVE) or triple sum frequency (TSF) spectroscopy,^77–79^ can control vibrational and vibronic coherences to investigate phonon–phonon and electron–phonon coupling mechanisms in greater detail. These approaches rely upon resolving the free-induction decay of vibrational modes involved in the vibrational or vibronic coherences of interest,^80^ which the HDFG experiments performed here show is possible (Figure 2).

In addition to resolving response from centrosymmetric materials, HDFG could be useful for investigating non-centrosymmetric materials. Unique polarization control available in hyper-Raman transitions can make HDFG a useful spectroscopy when performed alongside vSFG,^81,82^ which uses infrared and Raman transitions and is sensitive to symmetry breaking under the electric dipole approximation.^43,83^ Additionally, the sensitivity of hyper-Raman transitions to one- and two-photon allowed transitions may allow it to resolve peculiarities in the electronic structure of metal halide perovskites that are unavailable to other conventional techniques.^25,84–86^ Since HDFG is a weak process,^87^ one should take care to account for potential cascades from lower-order processes when considering HDFG response from noncentrosymmetric systems,^88,89^ as has been done in fifth-order coherent nonlinear spectroscopies.^12,90,91^ If HDFG could be isolated from cascades, it would be useful for investigating polarization-dependent vibrational response and electronic structure in noncentrosymmetric materials in tandem with vSFG.

In summary, preresonant hyper difference frequency generation (HDFG) spectroscopy is found to resolve coherent vibrational spectra and dynamics from FAPbBr_3_, a centrosymmetric metal halide perovskite. The coherent spectra and dynamics of a high-energy vibrational mode, important for understanding A-site cation hydrogen bonding in FAPbBr_3_, *ν*(NCN), were measured using HDFG spectroscopy. The measured time–frequency domain HDFG response from *ν*(NCN) resolved a dephasing time *T*_2_ = 0.8 ± 0.1 ps, in excellent agreement with FT-IR measurements, which motivates further development of resonant HDFG to probe electronic–vibrational coupling mechanisms in LHPs. Like resonance Raman and hyper-Raman spectroscopy of molecular systems, HDFG could be used as a potential probe of resonance trends in metal-halide perovskites involving high-energy vibrations of the A-site cations. By combining HDFG with other spectroscopic tools, further insight into the structural parameters that dictate the photophysics of hybrid inorganic–organic materials can be achieved. Overall, the HDFG approach discussed herein can be applied to a variety of centrosymmetric materials to resolve the vibrational dephasing dynamics of infrared-active vibrations and should motivate application of additional nonlinear spectroscopies to investigate coupling and symmetry-dependent transitions in LHPs.

## Experimental Section

The ultrafast spectrometer is described in detail elsewhere.^45^ An 80 MHz Ti-sapphire ultrafast oscillator (Spectra-Physics Tsunami) created 35 fs seed pulses, amplified by a 1 kHz regenerative amplifier (Spectra-Physics Spitfire Ace) at ∼800 nm. A mask in the regenerative amplifier stretched the input pulses to roughly 1 ps. The resultant output was used to pump four independent optical parametric amplifiers (OPAs), two of which are relevant for this study. OPA1 (TOPAS-800, Light Conversion), referred to as ω_1_, was tuned for difference frequency generation (DFG) with an external AgGaS_2_ stage (NDFG “DF1”, Light Conversion). OPA2 (OPA-800C, Spectra Physics), referred to as ω_2_, was tuned in its signal arrangement for output at 7700 cm^–1^. A 1000 nm long-pass filter (Thorlabs FGL1000) was placed on the ω_2_ beamline to minimize visible leakage. The OPAs were independently tuned and automated using the attune package and WinTOPAS software.^92^ The entire spectrometer was purged with dry air to minimize H_2_O induced pulse distortions.

To ensure frequency-dependent temporal pulse overlap, the maximum points of a third-order cross-correlation between ω_1_ and ω_2_ measured in CaF_2_ were used to account for frequency-dependent zero delay values.^92^ To ensure that all signals depend upon the infrared pulse, the ω_1_ beamline was chopped at 500 Hz. The phase-matched **k**_3_ = 2**k**_2_ – **k**_1_ output was directed into a monochromator (Horiba Micro-HR) to isolate the 2ω_2_ – ω_1_ frequency and detected on the intensity scale with a photomultiplier tube (Hamamatsu H7422-20). The analog output was recorded and converted to a digital output using a data acquisition unit (National Instruments). *In situ* power corrections were performed by dividing the recorded spectra by OPA output measured by custom-built pyroelectric detectors. Measurements were acquired using software controlled by the yaq and Bluesky packages.^93,94^ All data were collected at room temperature under ambient conditions and worked up using the open-source scientific Python stack.^95–97^

FAPbBr_3_ crystals were synthesized by adapting a previously reported crystal growth procedure for methylammonium-based 3D perovskite.^98^ Lead bromide (PbBr_2_) (CAS no. 10031-22-8), formamidinium bromide (FABr) (CAS no. 146958-06-7), γ-butyrolactone (GBL) (CAS no. 96-48-0), and dimethylformamide (DMF) (CAS no. 68-12-2) were used as received without further purification. Equimolar quantities of FABr and PbBr_2_ were dissolved in a 1:1 v/v DMF/GBL solution until it appeared homogeneous. The resulting solution was filtered using a polytetrafluoroethylene (PTFE) 0.2 μL syringe filter and transferred to a clean vial, which was heated to 80 °C. After 4 h at 80 °C, red perovskite crystals were obtained. These crystals were ∼1 mm in lateral dimension, allowing substrate-free mounting. Chlorobenzene was used to wash the 3D perovskite crystals, which were then immediately used in the nonlinear spectroscopy experiments. We note that while chlorobenzene has a broad combination band centered at ∼1736 cm^–1^,^99^ given its absence in the ATR-FT-IR spectrum, it has at most trace presence in our sample, so it is unlikely to contribute to the HDFG response. Infrared spectra were collected in attenuated total reflection (ATR) geometry (PIKE MIRacle) using a Bruker Tensor 27 spectrometer, controlled using OPUS 7.5.

## Supplementary Material



## Data Availability

The data and workup scripts that support this study are permissively licensed and are available on the Open Science Framework (OSF) at 10.17605/OSF.IO/PF46R.

## Notes

HDFG spectra of FAPbBr_3_ collected at ω_2_ = 7500 cm^–1^ (PDF)
