# Supplementary material for: Preresonant Coherent IR-hyper-Raman Spectroscopy of FAPbBr3
Source: J Phys Chem Lett. 2026 Sep 11;17(38):10895–902. doi: 10.1021/acs.jpclett.6c02047 (PMC13618031; doi:10.1021/acs.jpclett.6c02047)
Supplement: Supplementary file 1 [file jz6c02047_si_001.pdf]

Supporting Information for: Preresonant Coherent IR-hyper-Raman Spectroscopy of $FAPbBr_3$

Ryan P. McDonnell,[a] David P. Lafayette II,[a] Daniel D. Kohler, Jason M. Scheeler, Jalianet Román-Matías, Willa Mihalyi-Koch, Song Jin, and John C. Wright[b]
Department of Chemistry, University of Wisconsin - Madison, Madison, Wisconsin 53706, United States of America

CONTENTS

I. Supplementary Spectra S2

---

[a]These authors contributed equally to this work.
[b]Electronic mail: wright@chem.wisc.edu

I. SUPPLEMENTARY SPECTRA

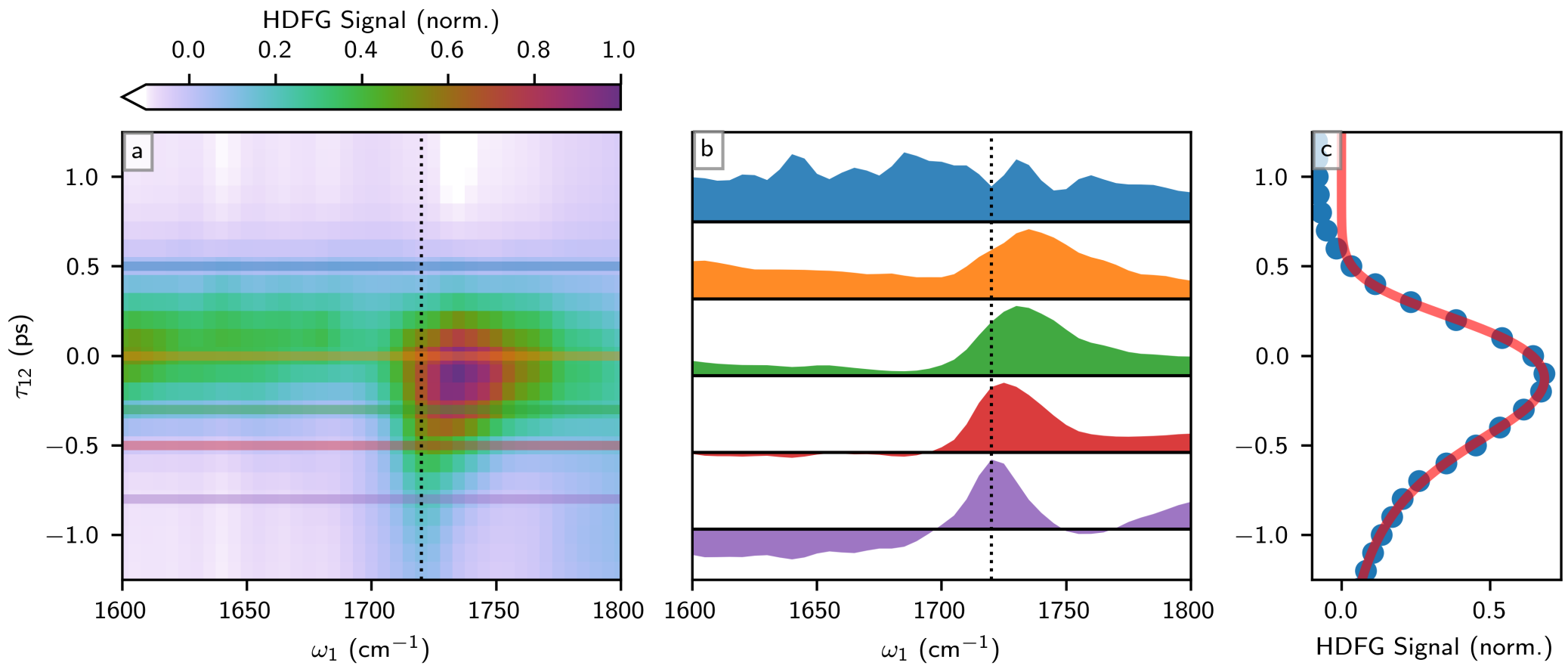

FIG. S1. HDFG response from single crystal $FAPbBr_3$, where the monochromator tracks output at $\omega_m = 2\omega_2 - \omega_1$ and ω_2 = 7500 cm^{-1}. (a): Frequency-time response in the 1600 - 1800 cm^{-1}region The spectrum in (a) has been smoothed and leveled to account for H_2O and other effects on pulse propagation, which results in "negative" intensities at some frequency and delay values. The two-photon interaction frequency is $2\omega_2$ = 15000 cm^{-1}. (b): 1D slices of the HDFG response in (a). Colors correspond to the horizontal slices in (a), indicating the time-delay. (c): Dynamics of the HDFG response at ω_1 = 1720 cm^{-1}. The thick red line overlaying the experimental response (blue dots) is a fit to equation 3 of the main text, with T_2 = 0.8 $\pm$ 0.1 ps. The static cutoff for $\tau_{12} > 0.5$ ps in the red trace arises from the Heaviside function used in the response function formalism (Equation 3 of the main text).
